# *Adenomatous polyposis coli* genotype-dependent toll-like receptor 4 activity in colon cancer

**DOI:** 10.18632/oncotarget.6844

**Published:** 2016-01-08

**Authors:** Feng Wen, Yongmei Liu, Wei Wang, Meng Li, Fuchun Guo, Yaxiong Sang, Qing Qin, Yongsheng Wang, Qiu Li

**Affiliations:** ^1^ The Department of Medical Oncology, Cancer Center, State Key Laboratory of Biotherapy/Collaborative Innovation Center for Biotherapy, West China Hospital, University of Sichuan, Sichuan, China; ^2^ State Key Laboratory of Biotherapy and Cancer Center/Collaborative Innovation Center for Biotherapy, West China Hospital, University of Sichuan, Sichuan, China

**Keywords:** adenomatous polyposis coli, NF-*κ*B, *β*-catenin, colon cancer

## Abstract

Toll-like receptors (TLRs)/NF-κB activation stimulated by lipopolysaccharide (LPS) was associated with diverse biological response in colon cancer, but the underlying mechanism was largely unknown. In the current study, we reported cell proliferation was elevated in adenomatous polyposis coli (APC) mutated- and APC knockdown cell lines, while the proliferation was inhibited in APC wild-type cell lines. Besides, *in vivo* experiments showed that LPS promoted APC knockdown tumor growth while inhibited proliferation of APC wild type. Further study confirmed that activation of TLRs/NF-κB signaling pathway by LPS cross regulated with APC/GSK-3β/β-catenin pathway, which were depend on APC status of cell lines. Taken together, APC genotypes play a key role in LPS induced different colon cancer biological response by cross-regulating β-catenin and NF-κB, which may provide a novel strategy for carcinogenesis prevention.

## INTRODUCTION

Colorectal cancer(CRC) is the fourth most common lethal malignancies with over one million new cases every year and is the second cause of cancer related death with more than 600,000 annually worldwide[1]. Though the treatment modalities for advanced CRC has been developed, including chemotherapy, radiation and targeted therapies, the over survivals are still suboptimal. Therefore, one of the most effective strategies in the control of CRC is the carcinogenesis prevention.

Nevertheless, the key factors attributing to the initiation of tumorigenesis are largely unknown. Recent studies have demonstrated that toll-like receptors (TLRs) play an important part at the beginning of carcinogenesis and development of CRC[2]. As we know, TLRs could mediate innate and adoptive immunity by activating transcription factors NF-κB following the stimulation of lipopolysaccharide (LPS), a specific component of gram-negative bacteria cell wall, such as Escherichia coli (E. coli) in intestinal[3, 4]. However, the role of TLRs in promoting or inhibiting tumor growth is confused. On one hand, in human colon carcinoma cells SW480, expression of TLR4 may increase immunosuppressive factors, promote apoptosis resistance and contribute to immune escape[5]. Meanwhile, some investigators showed in another human colon cancer cells SW620, TLR4 activation can trigger the proliferation and migration of cells[6]. Additionally, Hsu, R. Y. *et al.* showed that LPS-induced TLR4 signaling increases cell adhesion and liver metastasis in human colorectal cancer cells[7]. On the other hand, a recent study illustrated that TLR4 expression may be associated with mechanisms of preventing CRC progression [8]. In colon cell line HCT116, for example, activation of TLRs/NF-κB combined with radiation therapy can induce cell autophagy, which is a promising therapeutic strategy for enhancing radiation therapy [9].

To the best of our knowledge, adenomatous polyposis coli (APC) tumor suppressor pathway stands the dominant as a driven genetic alteration in colorectal tumorigenesis by aberrant signaling. Nearly 20% cases of colon cancer are associated with familial clustering, and familial adenomatous polyposis (FAP) is a well-defined genetic susceptibility to colorectal cancer[10–12]. However, the mechanisms that lies between FAP and colorectal cancer is indistinct, the tumorigenesis of which is the result of multiple factors. Among them, APC is a significant factor resulting in accumulation of β-catenin as well as oncogenes activation, including *c-Myc* and *CCND1*[13]. The mutation of APC is reported in approximately 85% sporadic or familial colorectal cancer, and is regard as an initial event of CRC related with cancer cell adhesion and proliferation[11, 14]. Hence, we supposed that APC may be related with the inconsistent influence of TLRs/NF-κB activation on CRC.

However, it merits further study to reveal the mystery about how APC affects TLRs/NF-κB activity. Indeed, the aberrant of APC determines the level of β-catenin in the multiprotein complex composed including axin and glycogen synthase kinase-3 (GSK-3β), which are the main members of Wnt signaling pathway. β-Catenin without phosphorylation in cytosolic associates with the TCF/LEF family, and works as a transcription factor to activate downstream responsive genes, playing a critical role in cancer development and body homeostasis [15].

Of particularly note, β-catenin plays a crucial role in colorectal tumorigenesis. Previously, Jiong Deng *et al.* illustrated that APC/GSK-3β/β-catenin pathway cross regulated NF-κB way through β-catenin and its target genes in colon cancer[16]. Meanwhile, Jiong Deng *et al.* also found that NF-κB activity was inhibited by the suppression of GSK-3β, whereas NF-kB activity was restored by re-expression of APC in APC mutated cells[17]. As a tumor suppressor gene, APC inactive mutations are considered as the predominant mechanism attributing to β-catenin deregulation. So we speculate that the cross regulation between TLRs and APC/GSK-3b/β-catenin may connect with APC gene mutation even in a feedback regulation.

Based on different APC genotypes, this study aims to investigate the function and regulatory mechanism of TLRs signal pathway in colon cancer growth, prove the connection of TLRs/NF-κB pathway activation and APC/GSK-3β/β-catenin, elucidate the influence of TLRs/NF-κB activation on tumor, and provide a new thread for colon cancer prevention.

## RESULTS

### Cell proliferation increased in APC aberrant colon cancers with the LPS stimulation

**Figure 1:**
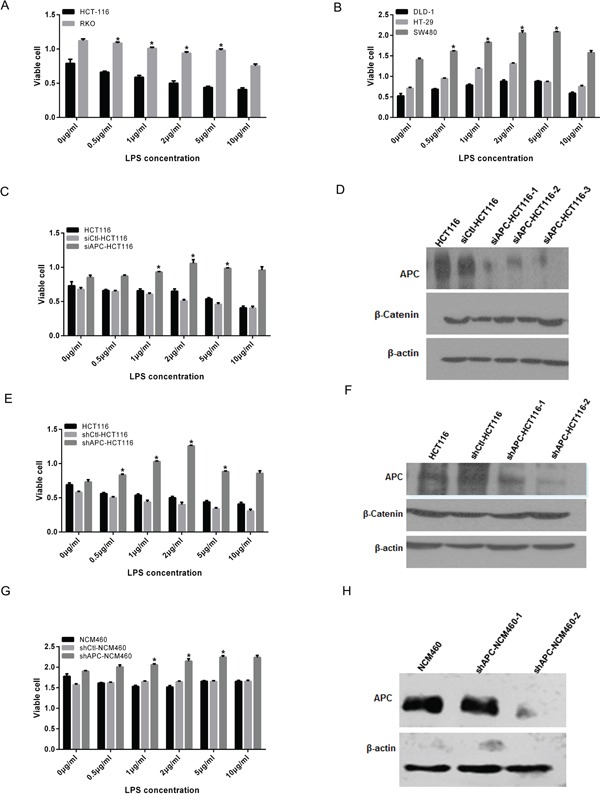
Cell proliferation increased in APC aberrant colon cancers with the LPS stimulation Different fold dilution series of LPS (0, 0.5, 1.0, 2.0, 5.0, and 10.0μg/ml) were added to the colon cell lines with different APC genotype for 24 hours, then MTT was applied to analyze the cell proliferation (*P < 0.05). **A.** HCT116 and RKO cell growth was inhibited especially in the concentration of 2.0, 5.0 and 10.0μg/ml. **B.** DLD-1, SW480 and HT29 increased significantly with the LPS stimulation. Additionally, proliferation of SW480 cell was most significant under the 2.0 μg/ml LPS stimulation. **C.** siCtl-HCT116 grew more slowly and significantly inhibited under the LPS stimulation. While in siAPC-HCT116, the growth rate was increased with the addition of LPS, especially in 2.0, 5.0 and 10.0μg/ml, the difference of which was statistically significant. **D.** Western blot confirmed that compared with siCtl-HCT116, the interference effect of siAPC HCT116-3 sequence was most obvious, and the levels of β-catenin elevated correspondingly. **E.** HCT116 and shCtl-HCT116 was inhibited with the LPS stimulation, while that of shAPC-HCT116 was accelerated, significantly at the concentration of 1.0, 2.0 and 5.0μg/ml. **F.** Western blot was showed that compared with shCtl-HCT116, APC levels of two interfering sequences were decreased obviously, and the levels of β-catenin elevated correspondingly especially the shAPC-HCT116-2. **G.** For shAPC-NCM460, a growth acceleration trend was observed in LPS stimulation compared with NCM460. **H.** Western blot confirmed the APC knockdown effect of stable shAPC-NCM460-2 cell line.

To verify the proliferation changes of colon cancer cells with different APC genotypes after the LPS stimulation, LPS with different fold dilution series (0, 0.5, 1.0, 2.0, 5.0, and 10.0 μg/ml) were added to the HCT116, RKO, SW480, DLD-1 and HT29 cultures. After 24 hours, MTT assay was applied to analyze the cell proliferation. Results showed that the proliferation of HCT116 and RKO cells were inhibited with the additional LPS, especially in the concentration of 2.0 μg/ml(Figure 1A), while SW480, HT29 and DLD-1 increased significantly with LPS stimulation (Figure 1B). Especially, the growth of SW480 was most significant under the 2.0 μg/ml LPS stimulation.

To investigate the characteristic of cells with specific biological changes after activation of TLRs/NF-κB, successfully transient interfering APC was performed in HCT116. Twenty-four hours after the disposal of siAPC, a red fluorescence microscope was sure an approximately 100% interference efficiency (sFigure 1A,1B). Simultaneously, HEK 293T cells were used to load lentivirus and the efficacy was shown in sFigure 1C. Western blot was applied to evaluate silencing efficiency of APC, and the results showed that compared with control, the interference effect of siAPC-HCT116-3 sequence was most obvious, and the levels of β-catenin elevated correspondingly after the interference of APC, which was shown in Figure 1D. To the stably knockdown of APC, APC levels of shAPC-HCT116-2 and shAPC-NCM460-2 were decreased obviously, and the levels of β-catenin elevated correspondingly, both of which were designated as APC mutation cell lines (Figure 1F, 1H).

As a negative control, siCtl-HCT116 grew more slowly and significantly inhibited under the LPS stimulation, while in siAPC-HCT116, the growth rate was increased with addition of LPS, especially in 2.0, 5.0 and 10.0μg/ml, the difference of which was statistically significant (Figure 1C, p <0.05). Similarly, cell proliferation of HCT116 and shCtl-HCT116 was inhibited with the LPS stimulation, while that of siAPC-HCT116 was accelerated, significantly at the concentration of 1.0, 2.0 and 5.0μg/ml (Figure 1E, p <0.05).

To analyze the LPS influence on normal intestinal epithelium, NCM460 and APC stable knockdown cells shAPC-NCM460 were applied. Cell growth of NCM460 showed a transient inhibition 24 hours after the LPS treatment, while for shAPC-NCM460, a growth acceleration trend was observed (Figure 1G). However, changes disappeared at 48 hours (sFigure 2A) and 72 hours (sFigure 2B). The growth of both cell lines slowed down in 72 hours and was independent to LPS stimulation, which might be related with the nature of normal cells.

### Apoptosis decreased in APC aberrant colon cancers with the LPS stimulation

**Figure 2:**
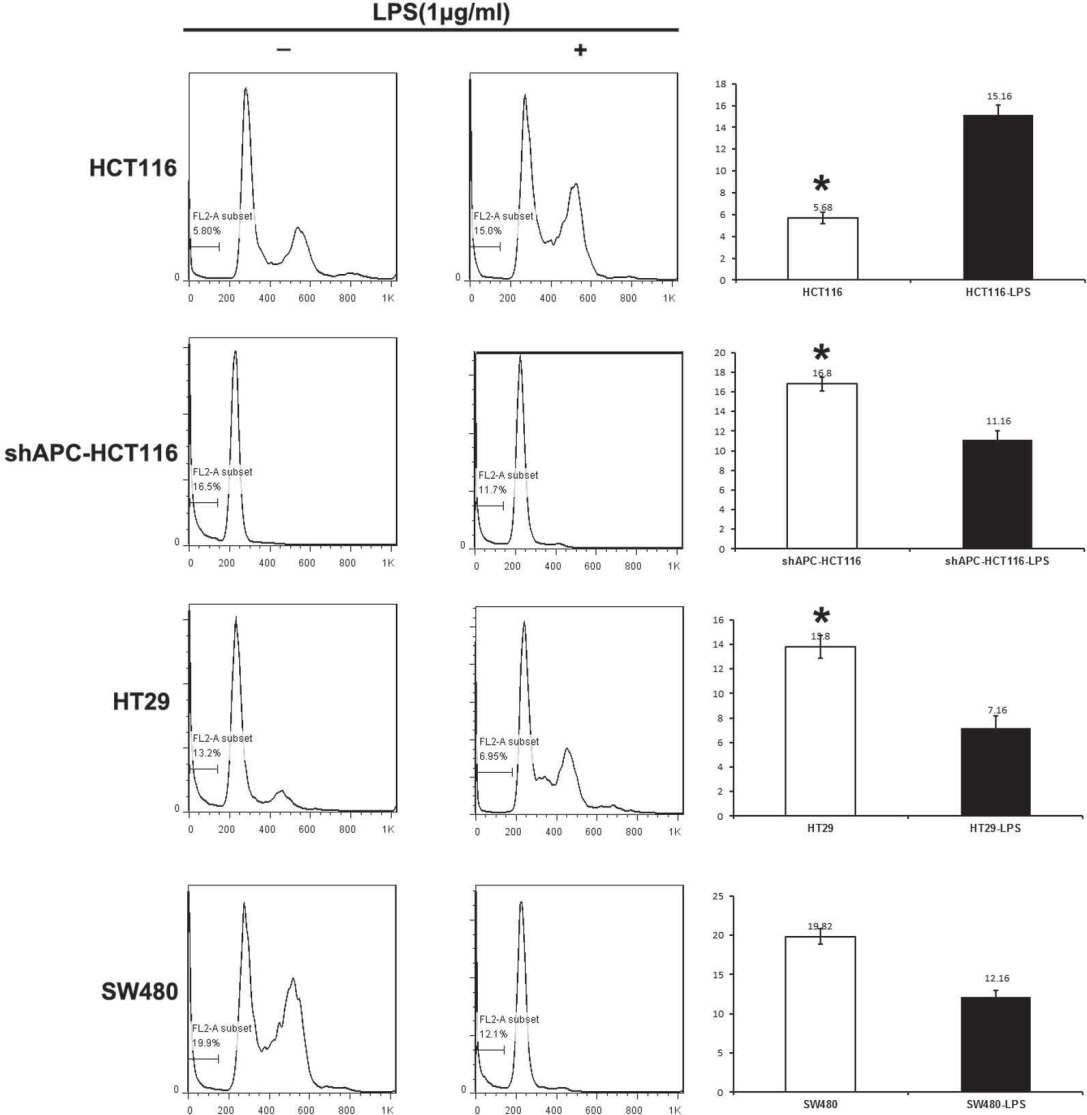
Apoptosis decreased in APC aberrant colon cancers with the LPS stimulation For cell apoptosis analysis, human colon cancer cell lines HCT116, ShAPC-HCT116, HT-29 and SW480 were disposed with 1.0μg/ml LPS and collected 24 hours later in 6-well plate.

For cell apoptosis analysis, HCT116, shAPC-HCT116, SW480 and HT29 were disposed with 1.0μg/ml LPS and collected 24 hours later in 6-well plate. The apoptosis of shAPC-HCT116 was declined from 16.6% to 11.7% with the additional LPS stimulation, from 13.2% to 6.95% in HT29, and from 19.9% to 12.1% in SW480. While the apoptosis ratio of HCT116 increased from 5.8% to 15.0%. All the apoptosis changes induced by LPS stimulation were significant except that of SW480 (Figure 2).

### The effect of LPS stimulation on HCT116 and shAPC-HC116 tumors *in vivo*

**Figure 3:**
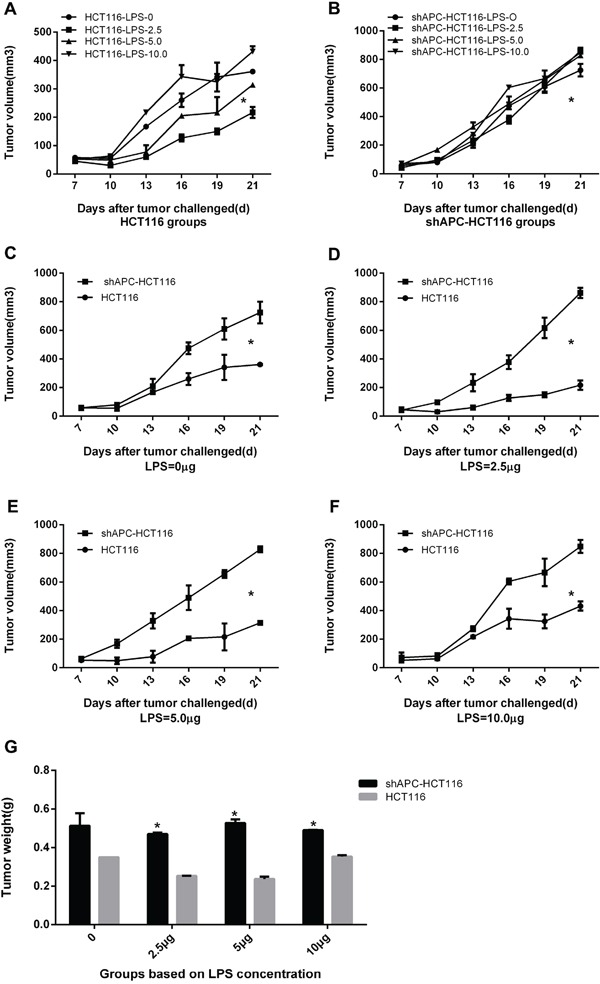
The effect of LPS stimulation on HCT116 and shAPC-HC116 *tumors in vivo* Vivo study was performed to explore the LPS stimulation effect in tumor models. **A.** Among four groups in HCT116 tumor-bearing mice, tumor growth of 2.5μg was slowest. **B.** For shAPC-HC116, compared with saline group, tumor growth of four LPS-stimulated groups were faster. **C, D, E** and **F.** Comparisons between HCT116 and shAPC-HC116 of four dose groups relatively, and the results showed that shAPC-HC116 grew faster in each group(*P< 0.05). **G.** Tumor weights of shAPC-HC116 were heavier compared with HCT116, especially in 0μg, 2.5μg, 5.0μg groups.

Furthermore, to test the effects of APC knockdown *in vivo*, HCT116 cells and shAPC-HCT116 cells were inoculated subcutaneously into the flanks of nude mice respectively. LPS of different concentrations were given by intratumoral injection. The treatment started 7 days after tumor inoculation, and repeated every three days for five times. Results showed that among four groups in HCT116 tumor-bearing mice, tumor growth of 2.5 μg was slowest (p <0.05) (Figure 3A). While for shAPC-HCT116, compared with saline group, tumor growth of four LPS-stimulated groups were faster (Figure 3B). Figure 3C, 3D, 3E and 3F were suggested that compared with HCT116 tumors, shAPC-HCT116 grew faster in each group and the differences were statistically significant (p <0.05 ). Meanwhile, tumor weights of shAPC-HCT116 were heavier compared with HCT116, especially in 0 μg, 2.5 μg, 5.0 μg groups, the differences of which were statistically significant (p <0.05; Figure 3G).

### Biological behavior verified with histologic analysis

**Figure 4:**
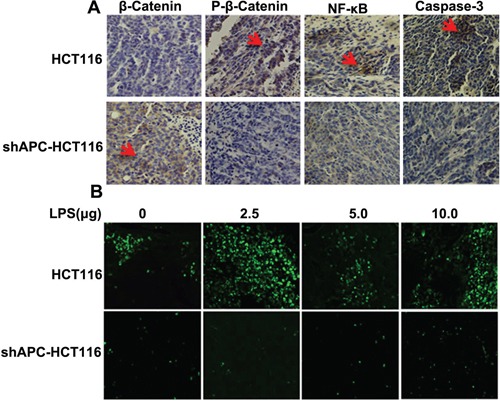
Biological behavior verified with histologic analysis **A.** β-catenin, p-β-catenin, NF-κB, and caspase-3 were evaluated by immunocytochemistry staining in HCT116 and shAPC-HCT116 tumors in LPS 2.5ug group; **B.** TUNEL was applied to analyze the apoptosis in tumor tissues, and compared with shAPC-HCT116, apoptosis of HCT116 were significant especially in the 2.5μg.

As we know, β-catenin mainly expressed in the cytoplasm inside, and appeared in the nucleus if activated. The immunocytochemistry staining of tumors showed that β-catenin level was lower in HCT116 than that of shAPC-HCT116 (Figure 4A). In terms of p-β-catenin, which mainly assembled in the cytoplasm without activity, higher p-β-catenin expression of HCT116 was seen than shAPC-HCT116 resulting from the normal β-catenin degradation complexity in HCT116. Consequently, high expression of β-catenin corrected with low level of caspase-3.

To analyze the apoptosis in tumor tissues, TUNEL was applied. Figure 4B showed that compared with shAPC-HCT116, apoptosis of HCT116 were more significant especially in the LPS 2.5 μg group, which were consistent with the results of tumor growth.

### Cross-regulation between β-catenin and TLR4/NF-κB

**Figure 5:**
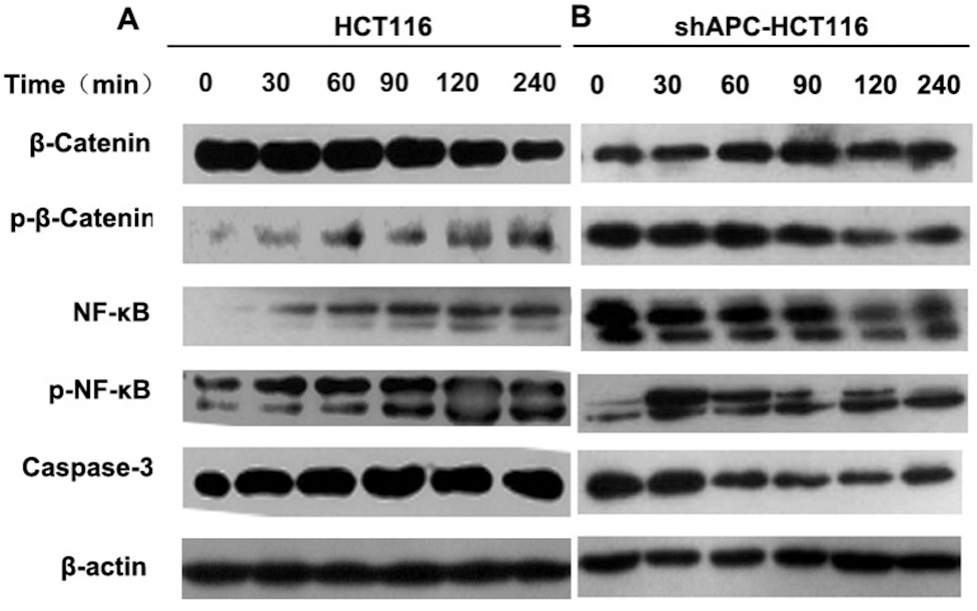
Cross-regulation between β-catenin and TLR4/NF-κB HCT116 **A.** and shAPC-HCT116 **B.** cells were stimulated with 1.0μg/ml LPS, and protein was collected at 30, 60, 90, 120 and 240 minutes. Western blot was applied to analyze the successive changes of β-catenin, NF-κB, p-β-catenin, p- NF-κB.

To further explore the mechanism of different biological behavior changes in different APC phenotype colon cancer cells with TLR4/NF-κB activation in vitro, proteins expression in tumor cells were valued. Figure 5A showed the successive changes of β-catenin and NF-κB within 240 minutes with 1.0μg/ml LPS stimulation in HCT116 cells. In detail, TLR4/NF-κB signaling pathway was activated after the LPS treatment, phosphorylated NF-κB (p-NF-κB) was increased correspondingly, which promoted the expression of β-catenin. Then the increased β-catenin was degraded in the wild-type APC cells leading to ascending of phosphorylation β-catenin. Eventually the β-catenin transcriptional function was inhibited. While cell apoptosis increased with the elevation of p-NF-κB resulting in raising of caspase-3 expression. On the country, the cross-regulation in shAPC-HCT116 was just the opposite. As shown in Figure 5B, the elevated β-catenin could not be fully degraded in shAPC-HCT116 cells. Hence, the NF-κB activity was inhibited by the elevated β-catenin leading to the decrease of p- NF-κB at the same time and the inhibition of caspase-3 activation as well. So the cell apoptosis was inhibited. Finally, the increased activated β-catenin into the nucleus played an important role as transcriptional factors initiating cell proliferation and differentiation.

To further verification of this mechanism, HT29 and SW480 were used (sFigure 3A and 3B). Similar with shAPC-HCT116, TLR4/NF-κB was activated by LPS stimulation. Increased NF-κB promoted β-catenin expression, which could not be degraded normally by APC complexity. As a reaction, the level of p-NF-κB was decreased by the elevated β-catenin. Finally, the cell proliferation was promoted, but cell apoptosis was inhibited.

### The β-catenin and NF-κB interaction *in vivo*

**Figure 6:**
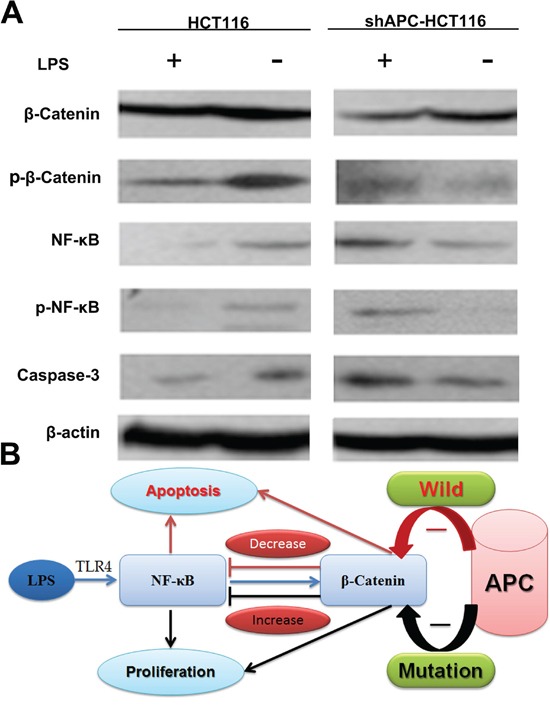
The β-catenin and NF-κB interaction *in vivo* **A**. Protein of tumor tissues was extracted to further validate the cross-regulation between TLR4/NF –κB and β-catenin in western blot. **B**. A flash of β-catenin and NF-κB interaction.

Protein of tumor tissues was extracted to validate the cross-regulation between TLR4/NF –κB and β-catenin in western blot. Figure 6 discovered an increase of NF-κB and activation of β-catenin expression in HCT116 groups by intratumoral injection of LPS, and the phosphorylation of β-catenin was increased because of the role of APC in degradation of β-catenin. Consequently, the proliferation of tumor cells was inhibited; meanwhile, NF-κB was activated, phosphorylation NF-κB was increased contributing to an increasing of caspase-3 and apoptotic cells. Hence, no significant increase of tumor growth was found in HCT116 mice. While in shAPC-HCT116 tumors, since the APC did not act normally in the degradation of β-catenin, the elevated β-catenin played as a transcriptional factor and eventually promoted the tumor growth. At the same time, NF-κB activity was inhibited by β-catenin, and p-NF-κB and caspase-3 expression were decreased. As a result, tumor cells proliferation was improved in shAPC-HCT116 mice.

## DISCUSSION

As one of the most common gastrointestinal malignancies, the occurrence and development of colorectal cancer are closely related to one's living habits. Approximately 85% colorectal cancer reported with APC protein mutation [16]. Though plenty studies have investigated the APC role in the colorectal cancer carcinogenesis, the relationship between APC mutation and intestinal bacteria stimulation is still not well recognized.

Additionally, some studies reported that TLR4-NF-κB activation played an important role in the development of tumors, which had caused widespread concern [19]. Some research showed that activation of TLR4 and NF-κB increased some chemokines expression, which induced cell apoptosis, inhibited tumor proliferation, and reduced tumor progression, resulting in a durability and effectiveness of anti-tumor immune response. Additionally, some studies reported that the activation of the signaling pathway may also increase the efficacy of cancer chemotherapy and radiotherapy [20–23]. Otherwise, it is reported that activation of TLR4 played an important role in the evolution of malignant behavior of tumor, including tumor formation, tumor invasion and distant metastasis [24].

In order to investigate the relationship between APC phenotypes and TLR4-mediated immune response in the development of colon cancer, we established stable APC knockdown cell lines, and to explore different biological behavior in different APC state under TLRs/NF-κB pathway activation, and to confirm that TLRs/NF -κB activation influences the development and progression of colorectal cancer in APC mutant population.

The current study showed that similarly to DLD-1, HT29 and SW480 with APC mutation, the cell proliferation was promoted both in siAPC-HCT116 and shAPC-HCT116 treated with LPS, while that was inhibited in APC wild type cell lines HCT116 and RKO. Western blot confirmed activation of TLRs/NF-κB signaling pathway by LPS cross regulated with APC/GSK-3β/β-catenin pathway differently in cells with different APC status and ultimately influenced cells apoptosis. *In vivo* experiments showed that LPS promoted shAPC-HCT116 tumor growth while inhabited tumor growth of HCT116, the difference of which was significant. Western Blot and immunohistochemistry confirmed the interaction between NF-κB and β-catenin in tumor tissue, which ultimately affected caspase-3 expression.

Based on the present study, we found cell proliferation of APC mutation colorectal cancer cells was accelerated with LPS stimulation. While in APC wild type cells, growth was suppressed and apoptosis was increased. The current findings were consistent with reports of Jiong Deng *et al.*[16]. Therefore, we found that the different biological effects of activating TLR4-NF-κB may depend on the APC/β-catenin signaling pathway, especially related with APC gene type.

Currently, it was reported that β-catenin was the downstream of NF-κB pathway, and played an important role in the gastrin-induced cell proliferation in colonic crypts[25]. Meanwhile, interaction of β-catenin and NF-κB signaling pathway was demonstrated to play an important role in the proliferation of intestinal inflammation [26]. Notably, Swidsinski *et al.* demonstrated the relationship between abundance of E. coli and CRC tumorigenesis early in 1998[27]. It is conceivable that intestinal microbiota plays a key role in the beginning of CRC with mounting evidence [28–31]. According to what had been founded in NCM460 and HCT116 cell lines, we suspected that in APC mutation population, ethanol was a risk factor for development of colorectal cancer, which might contribute to allowing gram-negative bacteria LPS into the blood, and activate TLR4/NF-κB signaling pathway. As a result of cross-regulation between NF-κB and β-catenin and APC abnormal function, level of β-catenin was increased leading to cell proliferation and apoptosis inhibition.

It is known to us that ethanol excessive consumption is proved to be a vital risk factor to colon cancer[32]. Though the ethanol itself is not defined as carcinogen, acetaldehyde, the direct oxidation product is considered as highly toxic and carcinogenic. Besides, it is reported that microorganisms can contribute to the production of acetaldehyde from ethanol and enhance its toxicity[33]. As the direct results, DNA is damaged and vitamin folate is degraded[33]. Meantime, the consumption of ethanol may impair the function of epithelium resulting in decreasing production of protective protein and antimicrobial peptides, which creates an opportunity for certain bacteria, for instance E. coli, to adhere to the colonic mucosa[34]. Of note, the chronic inflammation is a well-established risk factor for CRC[35]. There is no surprise that early study showed genetic events, including APC mutation and β-catenin activation may lead to barrier function loss in colonic epithelium and result in a translocation of microbial products into the tumor microenvironment[36].

To a certain extent, our results were the best supporting evidence of our hypothesis, which required further study of specific regulatory mechanisms. Nevertheless, the mechanisms are complex and largely unknown, which merits further study to reveal the mystery. While no particular bacteria have been proved to contribute the CRC, specific people with APC mutation are susceptible to the LPS from the intestinal bacterial based on our results, which is a promising preventive strategy. However, identifying risk population and introducing preventive intervention in CRC needs large scales of clinical trials.

In all, our study showed that changes of APC phenotype produced different biological behavior by regulating the levels of β-catenin, which interacted with NF-κB in activated TLR4-NF-κB pathway. Hence, intestinal microbiota/LPS stimulation was a risk factor for development of colorectal cancer in APC mutation population, which might provide a new strategy for the prevention of colorectal cancer.

## MATERIALS AND METHODS

### Cells lines and culture

HEK293 cells, human colorectal carcinoma cell lines HCT116, RKO, HT29, SW480, DLD-1 and normal intestinal epithelium cell line NCM460 were purchased from American Type Culture Collection (Rockville, MD)[18]. They were maintained in Dulbecco's modified Eagle's medium (Life Technologies, Inc) with 10% heat-inactivated fetal bovine serum (FBS) and antibiotics (100 U/ml penicillin and 100 mg/ml streptomycin) at 37°C in a humidified atmosphere containing 5% CO2.

### Small interfering RNA transfection

Small interfering RNA (siRNA) duplex oligo targeting human APC gene (three sequences were as follows:1-5′-CGAAATAGCTCCTCAAGTA-3′, 2-5′-CCAGAAGGCAATTGGAATA-3′, 3-5′-GCAAAGTCCTTCACAGAAT-3′) and a nonspecific duplex oligo as a negative control with fluorescence labeling (Ribobio, Guangzhou) were transfected at a concentration of 50nM with Lipofectamine (Invitrogen, US). Experiments were performed in 6-well plates.

### Lentivirus-mediated short hairpin RNA (shRNA) knockdown of *APC* expression

To knockdown APC expression and construct a stable cell line, pCDH-CMV-MCS-EF1-Puro (CD510B-1) shRNA vectors targeting human APC was purchased from Open Biosystems (Thermo Fisher Scientific, US). The sequences were based on the previous siRNA study. Lentiviral shRNA was produced by co-transfection of the trans- lentiviral packaging system by adding mixture of pCDH-CMV-MCS-EF1-Puro (CD510B-1)-shAPC and packaging plasmids pSPAX2 and pMD2.0G into HEK293T packaging cells (Open Biosystems, US). Viral supernatants were collected for cell infection, supplemented with 6 μg/mL polybrene. After 24 hours incubation, HCT116 and NCM460 were transduced by the lentiviral particles followed by puromycin selection (1.5μg/mL) for 7 days. The cells stably expressing shRNA were maintained in puromycin (0.75 μg/mL).

### Protein extraction and western blotting

To analyze protein expression in cells, human colon cancer cell lines were collected and washed with cold PBS. Subsequently cells were lysed in cold RIPA lysis buffer and protease inhibitors cocktail (Roche, US), which was performed on ice for 30 minutes. The supernatant of protein extracts were collected after centrifugation for 15 minutes at 4°C.

The concentrations of targeted protein were evaluated with the application of Bio-Rad protein assay reagent (Bio-Rad, US). Every simple about 20–40 mg was added to the lane of SDS polyacrylamide gel electrophoresis (SDS-PAGE) with loading buffer was loaded per lane, and the concentration of SDS-PAGE was 12%, except 6% for APC. The proteins were transferred to PVDF membrane filters (Millipore, US). Nonspecific binding was blocked with tris-buffered saline (TBS) containing 0.1% Tween 20 (TBS-T) and 5% skim milk for 2-5 hours at room temperature. The primary antibody (anti-human APC, β-catenin, p-β-catenin, NF-κB, p-NF-κB and caspase-3, anti-β-actin, 1:1000) were added to the PVDF membrane and incubated overnight at 4°C incubation. Then HRP-labeled secondary antibody (goat anti-rabbit 1: 5000, goat anti-mouse IgG 1: 10000) were incubated correspondingly for 1hour at 37°C. ECL chemiluminescence reaction solution was added to the membrane, and immune reactive bands were visualized using X-ray films.

Antibodies to NF-κB, p-NF-κB and caspase-3 were form Santa Cruz Biotechnology, US; and Antibodies to APC β-catenin and p-β-catenin were from Abcan, US.

### Cell proliferation assay

Different fold dilution series of LPS (E.coli O55: B5, sigma, US) were added to the human colon cancer cell lines HCT116, HT-29, SW480, DLD-1, RKO and NCM460. After 24 hours and 48 hours, 3-(4, 5-dimethylthiazol-2-yl)-2, 5-diphenyl tetrazolium bromide (MTT, Sigma Aldrich, Germany) was applied to analyze the cell proliferation which was quantified with colorimetric assay (EZ4U kit; Biomedica, Vienna). 570 nm of absorbance was recorded in a microplate reader. Experiment was repeated 3 times and results were the average of 6 wells.

### Flow cytometric apoptosis analysis

For cell apoptosis analysis, human colon cancer cell lines HCT116, shAPC-HCT116, HT-29 and SW480 were disposed with 1ug/ml LPS and collected 24 hours later in 6-well plate. Cells were harvested using cold PBS and fixed in 70% cold ethanol overnight in 4°C and treated with 1 ng/ml RNase A for 10 minutes at 37°C. Cellular DNA was stained with 15 ng/ml propidium iodide (PI, Sigma Aldrich, Germany) for 30 minutes at 37°C in the dark. The cells then were sorted by FACS Calibur Flow Cytometer (Becton Dickinson, US). The experiments were repeated three times.

### TUNEL assay

Fluorescent in situ terminal deoxynucleotidyltransferase-mediated nick end labeling (TUNEL) assay was performed using an in situ apoptotic cell detection kit (Boehringer Mannheim, US) following the manufacturer's protocol. To block the endogenous peroxidase, 1.3% H_2_O_2_ in PBS was used for 10 min at room temperature before enzymatic labeling. Then samples were washed by PBS. Anti-fluorescence antibody with peroxidase was added for 30 minutes. And chromogen 3,3′-diaminobenzidine tetrahydrochloride (DAB, Sigma-Aldrich, Germany) was applied to final color reaction.

### Immunocytochemistry staining

Tumor tissues were deparaffinized and then disposed with a gradient of alcohol (100%, 95%, 80% and 70%) and then washed five times. They were subjected to 0.05% trypsin in PBS and then disposed with 0.3% H_2_O_2_ in methanol. Subsequently, tissue samples were subjected to 10% horse serum for 30 min. Then they were incubated with different primary antibodies overnight respectively (β-catenin, p-β-catenin, NF-κB, and caspase-3, 1:100 dilution). Then secondary antibodies bio- anti mouse and rabbit IgG (1:200 dilution) were incubated with the sections for 1 hour and then incubated with avidin biotin-peroxidase complex diluted in PBS. Finally, tissues were stained with DAB and observed under light microscope.

### *In vivo* studies of animal tumor models

Six to eight week-old, 18-24 g of female nude mice were injected subcutaneously into right flank with untransfected cells HCT16, or shAPC-HCT116 cells (5 × 10^6^ cells in 100 μl PBS) to establish tumors. The tumor-bearing nude mice model of HCT116 cells and shAPC-HCT116 randomly divided into four groups respectively. LPS 2.5μg, 5.0μg and 10.0μg were given by intratumoral injection, and saline 100μl were given to the control groups. The treatment started 7 days after tumor inoculation, and repeated every three days for five times. Tumor mass and volume were measured every three days before the treatment. Three days after the last treatment, mice were sacrificed, and tumors were harvested.

All studies involving mice were approved by the institute's Animal Care and Use Committee.

### Data analysis and statistics

Analysis of variance (One-Way ANOVA) was performed to compare the individual time points among groups and Student's t-test was applied to analyze the differences between two groups. Statistical significance was set at P<0.05.

## SUPPLEMENTARY FIGURES



## Abbreviations

CRC: colorectal cancer
TLRs: toll-like receptors
LPS: lipopolysaccharide
shRNA: short hairpin RNA
UTR: untranslated region
qRT-PCR: quantitative real time PCR
MTT: 3-(4,5-dimethylthiazol-2-yl)-2,5-diphenyltetrazolium
FACS: fluorescence-activated cell sorting
PI: propidium iodide
PBS: phosphate-buffered saline
APC: adenomatous polyposis coli
NF-κB: Nuclear factor κB
FAP: adenomatous polyposis
GSK-3β: glycogen synthase kinase-3
TUNEL: terminal deoxynucleotidyltransferase-mediated nick end labeling
DAB: 3,3 -diaminobenzidine tetrahydrochloride
