# Supplementary material for: Adenomatous polyposis coli genotype-dependent toll-like receptor 4 activity in colon cancer
Source: Oncotarget. 2016 Jan 8;7(7):7761–72. doi: 10.18632/oncotarget.6844 (PMC4884952; doi:10.18632/oncotarget.6844)
Supplement: Supplementary file 1 [file oncotarget-07-7761-s001.pdf]

SUPPLEMENTARY FIGURES

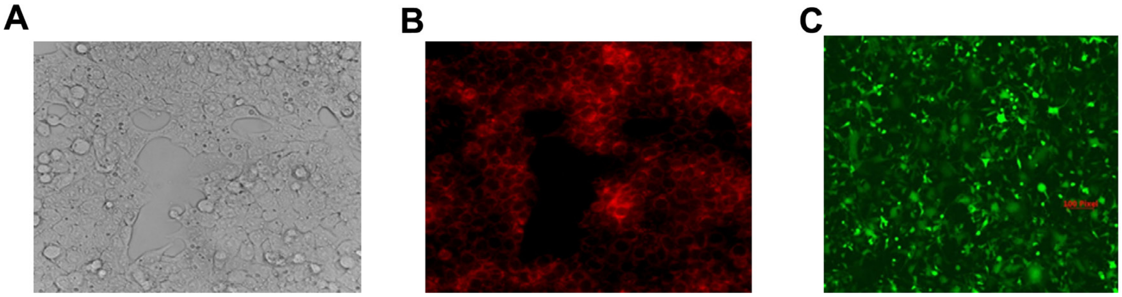

Supplementary Figure S1: The efficacy of APC knockdown in colon cancer. **A, B.** Twenty-four hours after the disposal of siAPC, a red fluorescence microscope was shown with approximately 100% interference efficiency; **C.** HEK 293T cells were used to pack of lentivirus efficiently.

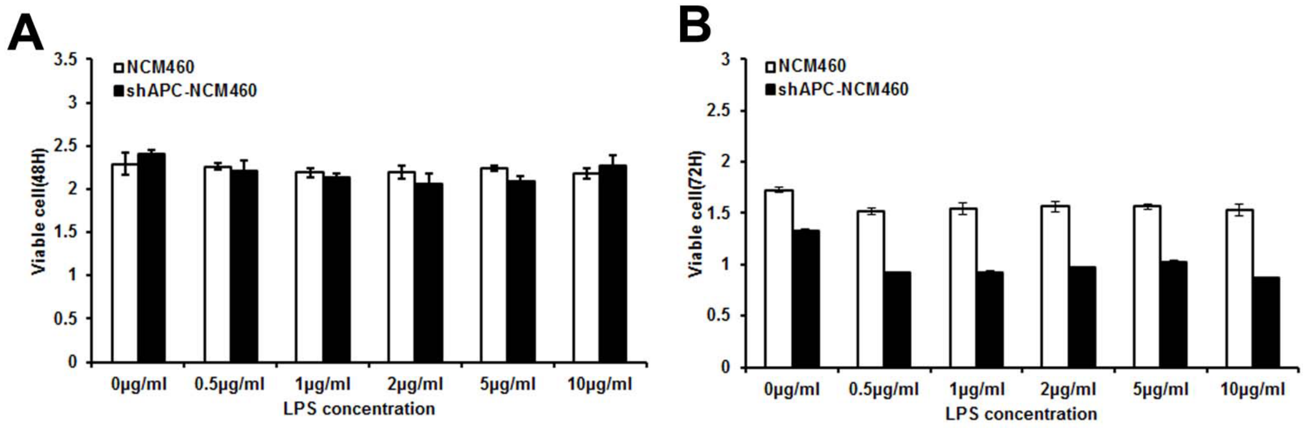

Supplementary Figure S2: The proliferation of shAPC-NCM460 in 48 and 72 hours with LPS stimulation. Different fold dilution series of LPS were added. After 48 hours and 72 hours, MTT was applied to analyze the cell proliferation, and no statistical differences were found.

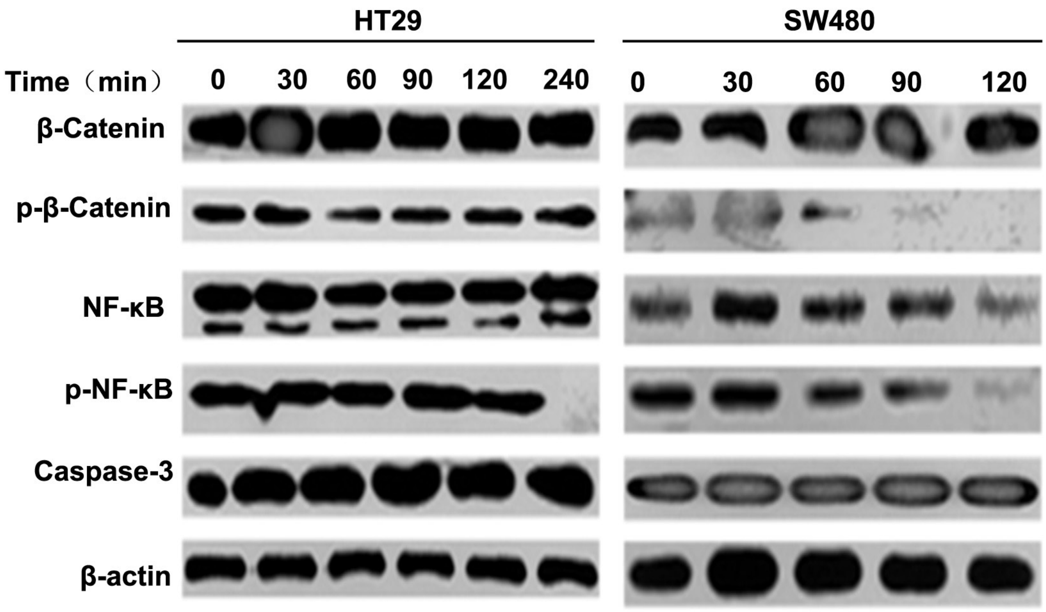

Supplementary Figure S3: The β-catenin and NF-κB interaction in HT29 and SW480. Western blot was applied to analyze the successive changes of β-catenin and NF-κB within 240 minutes with 1.0μg/ml LPS stimulation in HT29 and SW480.
